# Health literacy of pregnant women attending antenatal care clinics in Mansoura district, Egypt

**DOI:** 10.1186/s42506-022-00119-z

**Published:** 2022-12-01

**Authors:** Noha Essam, Mohamad Azmy Khafagy, Doaa Shokry Alemam

**Affiliations:** Department of Public Health and Community Medicine, Faculty of Medicine, Mansoura University, El-Gomhouria Street, El-Mansoura, PO 35516 Egypt

**Keywords:** Health literacy, Pregnant women, Egypt

## Abstract

**Background:**

Health literacy (HL) is an important maternal factor that is involved in the engagement of a mother and her children with health promotion and preventive activities. Studies have found poor HL in large proportions of the population of both developed and developing countries. This study measures the HL of pregnant women and explores its associated factors.

**Methods:**

A cross-sectional study was conducted on 382 pregnant women attending antenatal care clinics in Mansoura district, Egypt, using an interviewer-administered questionnaire. The Arabic version of the European Health Literacy Survey Questionnaire-short version (HLS-EU-Q16) was used to assess the HL of the participants.

**Results:**

The study showed that 79.8% of studied pregnant women had limited HL (34.5% insufficient HL and 45.3% problematic HL), and only 20.2% of them had sufficient HL. Limited HL was independently predicted by unsatisfactory income (*OR* = 6.9; 95% confidence interval [CI]: 3.2–15.3; *P* ≤ 0.05), lower than university education (*OR* = 5.3; 95% *CI*: 1.6–17.2; *P* ≤ 0.05), and having unplanned pregnancy (*OR* = 3.7; 95% *CI*: 1.6–8.5; *P* ≤ 0.05).

**Conclusion:**

The majority of pregnant women in this study had limited HL. It was more frequent among women with lower levels of education, insufficient incomes, and unplanned pregnancies. Antenatal care programs should provide services that respond to the HL level and needs of pregnant women.

## Introduction

Health literacy (HL) is an integral aspect of health promotion and communication. Higher levels of HL are not necessarily tied to years of study. HL is a set of skills, such as analysis, decision-making, and the ability to use knowledge in health situations [1].

The World Health Organization defines HL as the cognitive and social skills that determine the motivation and ability of individuals to gain access to, understand, and use information in ways that promote and maintain good health [2]. HL thus concerns the capacities of people to meet the complex demands of maintaining health in modern society [3]. Three important domains make up an individual’s HL: the functional, comprehensive, and critical domains. Functional HL refers to reading and writing skills, which facilitate effective health-related performance in everyday activities. Comprehensive HL refers to advanced skills that facilitate active contributions in everyday situations, obtaining and interpreting information from different sources and using new information in response to changing circumstances. Critical HL is related to the ability to make informed health decisions in the context of everyday life at home, in the community, at the workplace, and in the healthcare system [4].

Studies have shown that having a low level of HL has negative consequences for one’s health and for the uptake of health system services. Low HL is associated with lower levels of use of preventive services, greater use of emergency services, poorer adherence to medication regimens, poorer health outcomes, and higher risk of hospitalization [5, 6]. Enhancing HL helps improve the utilization of services, behaviors, and self-care [7, 8].

It is important to understand HL in the context of pregnancy for two reasons. First, pregnancy may be the “entry point” to health care [9]. Second, a woman’s level of HL may not only affect her own health but also her children, before conception, during pregnancy, and during their formative years [10].

Low maternal HL is associated with omission of preconception counseling, delayed initiation of antenatal care (ANC), and missed antenatal visits. Moreover, low HL was also associated with inadequate antenatal health behaviors and poorer obstetric and postpartum outcomes [11, 12].

Although low HL is common in developed countries [13–15], the level of HL among Egyptian people has not been sufficiently well described, and few studies have identified its significant aspects. A study at the Ain Shams University Hospitals showed that 75% of elderly caregivers had low HL [16]. Another study found that more than 80% of the attendees of the outpatient clinics at the El-Demerdash University Hospital in Cairo had low HL [17]. Research on HL among pregnant women in Egypt is limited. This study aims to measure the HL level of pregnant women and to explore its associated factors.

## Methods

### Study design and setting

A cross-sectional study was carried out in the “Mansoura district,” which is one of 18 districts in the Dakahlia Governorate Egypt. It includes the city of Mansoura and the surrounding 57 villages. This study was carried out from July 2019 to March 2020. Participant recruitment took place in the ANC clinics in two settings:

1. Primary healthcare (PHC) facilities: two rural PHC facilities, namely, Shawa family medicine center and Met-Khames family medicine unit, and two urban PHC facilities, namely, the Kolongel family medicine unit and the El-Ferdous health unit.
2. Mansoura University Hospital (MUH), in particular, the ANC clinics of obstetrics and gynecology department in MUH, the largest tertiary level referral teaching hospital in Mansoura

### Study population and sampling method

The study targeted pregnant women in the second and third trimesters. Inclusion criteria were being an Egyptian, able to communicate, and residing in the Mansoura district. Pregnant women with degrees in the medical sciences were excluded. The required minimum sample size was calculated as 237 participants, using the following formula [18].

$$n=\frac{Z^2\ P\left(1-P\right)}{d^2}$$

where

*Z* = 1.96 for 95% confidence interval

*P* = expected prevalence of low HL (81%) [17]

*d* = precision (margin of error) = 0.05

The sample size was increased by about 10% as a buffer against missing or incomplete data. The final sample was 400 participants to compensate for nonresponses. The population of Mansoura district is 49% urban and 51% rural residents. We selected a sample with 50% of participants (*n* = 200) from the PHC level, which mainly serves rural residents, and the other 50% (*n* = 200) from secondary and tertiary health care, which serves both rural and urban residents. The following methods were followed to obtain the predetermined sample size from the study settings:

A. From PHC facilities: A two-stage random sampling technique was adopted. In the first stage, two urban and two rural PHC facilities were selected from a list of 54 PHC facilities in Mansoura district provided by the district health office. In the second stage, a sample of 200 pregnant women (50 pregnant women from each predetermined facility) was selected using a systematic random sampling technique, selecting each fifth pregnant woman arriving at the clinic.
B. From the ANC clinics in MUH, a sample of 200 pregnant women was recruited using a systematic random sampling technique, selecting each fifth pregnant woman attending the clinic.

**Fig. 1:**
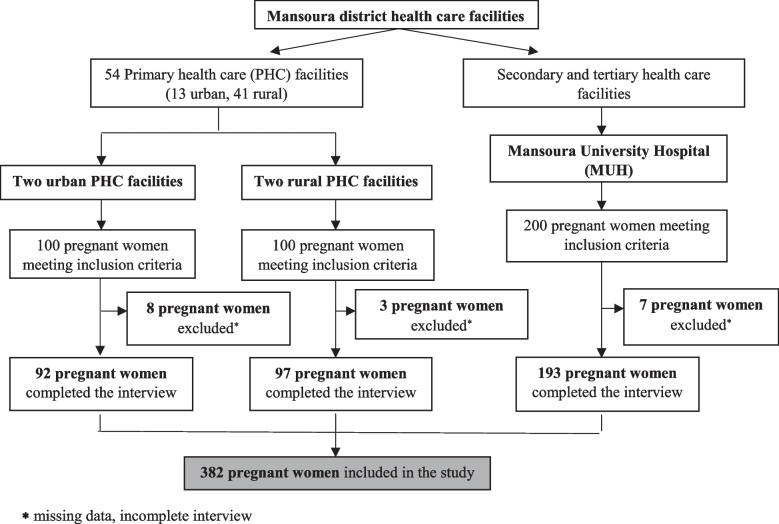
Flow chart of participants’ sample. *Missing data, incomplete interview

About 95.5% (*n* = 382) of the sample completed the questionnaire (Fig. 1).

### Study tools

The data were collected using a structured questionnaire composed of three parts. Part 1 collected sociodemographic characteristics of pregnant women (10 questions including a single question about family income). Part 2 collected the obstetric characteristics (10 questions). Part 3 included the European Health Literacy Survey Questionnaire—short version (HLS-EU-Q16) to assess HL. It consisted of 16 questions (e.g., how easy is it to understand what your doctor says to you?/how easy is it to judge if the information on health risks in the media is reliable?) Each question featured the same four response categories: very easy, easy, difficult, and very difficult [19]. The response categories were dichotomized into easy (given a value of 1) and difficult (given a value of 0). A sum score of the response values was calculated to give a total HL score ranging from 0 to 16. The HL scores were divided into three categories: inadequate HL (0–8 points), problematic HL (9–12 points), and sufficient HL (13–16 points). To compare the groups, insufficient and problematic HL were grouped as limited HL for comparison with sufficient HL.

The questionnaire was developed in English. It was translated into Arabic by using a back-translation method. The content validity of the questionnaire was ensured by a consulting panel of experts and amended according to their comments and suggestions. Finally, the Arabic version was tested on a group of 40 pregnant women (not included in the full-scale study) to clarify any difficulties with the questionnaire, flow of work, or administrative cooperation. Some questions were modified, while others were amended to facilitate their use and interpretation. To test for reliability, the Cronbach’s alpha coefficient for the Arabic version of the HLS-EU-Q16 scale was computed to *α* = 0.761, an acceptable value. The data were collected using a face-to-face interview. The respondents took approximately 15–20 min to complete the questionnaire.

### Statistical analysis

The collected data were coded, processed, and analyzed using the Statistical Package of Social Science program for Windows (version 16) (Chicago, IL, USA). The categorical data were presented as numbers and percentages. Chi-square and Fisher’s exact tests were used to test the significance of the associated categorical variables. The significance of the results was judged at *P* ≤ 0.05.

The significant variables on univariate analysis were entered into the logistic regression model using the forward Wald statistical technique to predict the most significant determinants and control for possible interactions and confounding effects. In the regression model, HL was entered as the dependent variable, and the independent variables included family income, education, and pregnancy planning. Adjusted odds ratios and their 95% confidence intervals (CIs) were calculated.

## Results

A total of 382 pregnant women with a mean age of 27.1 ± 5.5 years participated in the study. The majority of the participants had a rural residence (60.7%), had a lower than university education (77.8%), were housewives (78.8%), had unsatisfactory income (53.4%), and were not suffering from any chronic diseases (83%) (Table 1).

**Table 1:** Sociodemographic characteristics of the studied pregnant women attending antenatal care (ANC) clinics in Mansoura district, Egypt, 2020 (*n* = 382)

Sociodemographic characteristics	Total (*n* = 382) *N* (%)
Age	
< 20	22 (5.8)
20	214 (56)
30	138 (36.1)
≥ 40 years	8 (2.1)
Mean ± SD	27.1 ± 5.5
Residence	
Urban	150 (39.3)
Rural	232 (60.7)
Educational attainment	
< University education	297 (77.8)
≥ University education	85 (22.2)
Working status	
Housewife	301 (78.8)
Working	81 (21.2)
Family income	
Unsatisfactory	204 (53.4)
Satisfactory	178 (46.6)
Suffering from chronic disease	
Yes	65 (17)
No	317 (83)

**Fig. 2:**
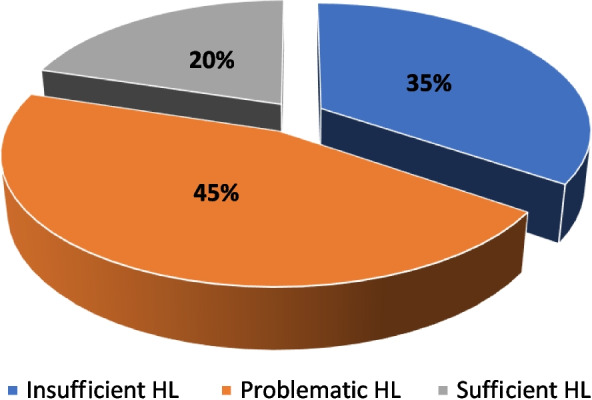
Health literacy (HL) level of studied pregnant women attending antenatal care (ANC) clinics in Mansoura district, Egypt, 2020 (*n* = 382)

The HL levels of the participants were unfavorable, with 79.8% having limited HL (34.5% insufficient HL and 45.3% problematic HL), while only 20.2% have sufficient HL (Fig. 2).

The risk of limited HL is higher among younger women (*OR* = 2.2; 95% *CI*: 1.32–3.72; *P* ≤ 0.05), less than university educated women (*OR* = 7.9; 95% *CI*: 4.54–13.80; *P* ≤ 0.05), housewives (*OR* = 2.1; 95% *CI*: 1.22–3.74; *P* ≤ 0.05), and women with unsatisfactory incomes (*OR* = 10.3; 95% *CI*: 5.24–20.40; *P* ≤ 0.05) (Table 2). In addition, HL has statistically significant associations with husband’s educational attainment, such that the risk of limited HL is higher among women whose husbands have a below-university level education (*OR* = 4.9; 95% *CI*: 2.86–8.34; *P* ≤ 0.05). In addition, women whose most dependable source of health information is not the Internet show a significant risk for limited HL (*OR* = 3.5; 95% *CI*: 2.0–6.1; *P* ≤ 0.05). On the other hand, HL had no statistically significant relation to residence, husband’s age, husband’s working status, or the presence of chronic disease (Table 2).

**Table 2:** Relationship between level of health literacy (HL) and sociodemographic characteristics of pregnant women attending ANC clinics in Mansoura district, Egypt, 2020 (*n* = 382)

Sociodemographic characteristics	Limited HL (*n* = 305) *N* (%)	Sufficient HL (*n* = 77) *N* (%)	Test of significance χ^2^	COR (95% *CI*)
Age^(a)^				
≤ 27 years	166 (86)	27 (14)	*P* ≤ 0.05*	2.2 (1.32–3.72)
> 27 years	139 (73.5)	50 (26.5)		1 (r)
Residence				
Urban	117 (78)	33 (22)	*P* > 0.05	1 (r)
Rural	188 (81)	44 (19)		1.2 (0.73–2.00)
Educational attainment				
< University education	263 (88.6)	34 (11.4)	*P* ≤ 0.05*	7.9 (4.54–13.80)
≥ University education	42 (49.4)	43 (50.6)		1 (r)
Working status				
Housewife	249 (82.7)	52 (17.3)	*P* ≤ 0.05*	2.1 (1.22–3.74)
Working	56 (69.1)	25 (30.9)		1 (r)
Husband’s age^(a)^				
≤ 32 years	164 (80)	41 (20)	*P* > 0.05	1.0 (0.62–1.69)
> 32 years	141 (79.7)	36 (20.3)		1 (r)
Husband’s educational attainment				
< University education	252 (86.9)	38 (13.1)	*P* ≤ 0.05*	4.9 (2.86–8.34)
≥ University education	53 (57.6)	39 (42.4)		1 (r)
Husband’s occupation				
Not working	4 (80)	1 (20)	FET	1 (0.11–9.17)
Working	301 (79.8)	76 (20.2)	*P* > 0.05	1 (r)
Family income				
Unsatisfactory	193 (94.6)	11 (5.4)	*P* ≤ 0.05*	10.3 (5.24–20.40)
Satisfactory	112 (62.9)	66 (37.1)		1 (r)
Suffering from chronic disease				
Yes	49 (75.4)	16 (24.6)	*P* > 0.05	1 (r)
No	256 (80.8)	61 (19.2)		1.4 (0.73–2.57)
Most frequent source of health information				
Internet	43 (60.6)	28 (39.4)	*P* ≤ 0.05*	1 (r)
Other sources	262 (84.2)	49 (15.8)		3.5 (2.0–6.1)

*r* reference group, *COR* crude odds ratio, *CI* confidence interval, *FET* Fisher exact test

*Statistically significant difference

^(a)^ categories based on median age. χ^2^, chi-square test

HL showed a significant association with pregnancy planning, age at marriage, and age at first child. The risk of limited HL was higher among women with unplanned pregnancy (*OR* = 2.9; 95% *CI*: 1.51–5.42; *P* ≤ 0.05), younger age at marriage (*OR* = 5.7; 95% *CI*; 3.22–10.20; *P* ≤ 0.05), and younger age at first child (*OR* = 8.3; 95% *CI*: 4.46–15.56; *P* ≤ 0.05) (Table 3). HL also shows a significant association with abortion history and health problems in previous pregnancies. A higher risk for limited HL is found among women without a history of abortion (*OR* = 1.7; 95% *CI*: 1.04–2.89; *P* ≤ 0.05) and those reporting health problems during last pregnancy (*OR* = 1.7; 95% *CI*; 1.04–2.88; *P* ≤ 0.05) (Table 3).

**Table 3:** Relationship between limited comprehensive HL and current obstetric characteristics of studied pregnant women attending ANC clinics in Mansoura district, Egypt, 2020 (*n* = 382)

Obstetric characteristics	Limited HL (*n* = 305) *N* (%)	Sufficient HL (*n* = 77) *N* (%)	Test of significance χ^2^	COR (95% *CI*)
Gestational age				
Second trimester	101 (77.1)	30 (22.9)	*P* > 0.05	1.3 (0.77–2.16)
Third trimester	204 (81.3)	47 (18.7)		1 (r)
Gravidity				
Prim gravida	67 (84.8)	12 (15.2)	*P* > 0.05	1.5 (0.78–2.99)
Multigravida	238 (78.5)	65 (21.5)		1 (r)
Parity				
Nullipara	88 (83)	18 (17)	*P* > 0.05	1.3 (0.74–2.38%)
Multipara	217 (78.6)	59 (21.4)		1 (r)
Current pregnancy planning				
Planned	193 (75.1)	64 (24.9)	*P* ≤ 0.05*	1 (r)
Unplanned	112 (89.6)	13 (10.4)		2.9 (1.51–5.42)
Age at marriage^(a)^				
≤ 20 years	194 (91.5)	18 (8.5)	*P* ≤ 0.05*	5.7 (3.22–10.20)
> 20 years	111 (65.3)	59 (34.7)		1 (r)
Age at first child^(a)^				
≤ 21 years	198 (93.4)	14 (6.6)	*P* ≤ 0.05*	8.3 (4.46–15.56)
> 21 years	107 (62.9)	63 (37.1)		1 (r)
Abortion history				
Yes	99 (73.9)	35 (26.1)	*P* ≤ 0.05*	1 (r)
No	206 (83.1)	42 (16.9)		1.7 (1.04–2.89)
Health problems in previous pregnancies**				
Yes	65 (86.7)	10 (13.3)	*P* ≤ 0.05*	1.7 (1.04–2.88)
No	152 (75.6)	49 (24.4)		1 (r)
Previous labor method**				
Normal	39 (70.9)	16 (29.1)	*P* > 0.05	1 (r)
Cesarian	178 (80.5)	43 (19.5)		1.7 (0.87–3.32)
Previous labor site**				
Health facility	215 (78.5)	59 (21.5)	FET	
Home	2 (100)	0	*P* > 0.05	

*r* reference group, *COR* crude odds ratio, *CI* confidence interval, *FET* Fisher’s exact test

*Statistically significant difference

**Data analysis excluded 106 nulliparous women

^(a)^ categories based on median age. χ^2^, chi-square test

The logistic regression analysis and adjustment to the confounding factors showed that the following was significant independent predictors of limited HL: unsatisfactory income (*OR* = 6.9; 95% *CI*: 3.2–15.3; *P* ≤ 0.05), lower than university education (*OR* = 5.3; 95% *CI*: 1.6–17.2; *P* ≤ 0.05), and unplanned pregnancy (*OR* = 3.7; 95% *CI*: 1.6–8.5; *P* ≤ 0.05) (Table 4).

**Table 4:** Multivariate logistic regression analysis for determining the predictors of limited HL in pregnant women (*n* = 305) based on forward Wald method

Predictors	AOR (95% *CI*)	*p*-value^*^
Family income		
Unsatisfactory	6.9 (3.2–15.3)	*P* ≤ 0.05
Satisfactory	1 (r)	
Educational attainment		
< University education	5.3 (1.6–17.2)	*P* ≤ 0.05
≥ University education	1 (r)	
Current pregnancy planning		
Planned	1 (r)	*P* ≤ 0.05
Unplanned	3.7 (1.6–8.5)	
Constant	1.377	
% Predicted	84.8%	
Model χ^2^	122.515	
−2 log likelihood	261.450	

*r* reference group, *AOR* adjusted odds ratio, *CI* confidence interval

*Statistically significant if *P* ≤ 0.05

## Discussion

HL is an important challenge for public health. Low HL has various negative consequences for both individuals and for society as a whole [20–22]. Understanding HL in the context of pregnancy is important because it can influence how women deal with the greater need for health information associated with pregnancy and ANC [10].

In the present study, the majority of participants had limited HL (79.8%). There were no available data on the rate of low HL among Egyptian pregnant women as a whole. However, our result is similar to that of an Egyptian study of outpatient attendees at the El-Demerdash University Hospital, which reported a high prevalence of limited HL (81%) [17].

Likewise, the results found here resemble the prevalence of limited HL (66.1%) found among pregnant Turkish women [23]. Similarly, more than three-quarters of pregnant women showed low HL in Iran and Laos [24, 25].

This study identified a significant association between the age of a pregnant woman age and her HL level: younger women had lower HL. Young women are usually less experienced with health issues in general, which might explain this finding. This result is in agreement with results of some Iranian studies in which increased age was associated with higher HL level [26, 27]. By contrast, a study in Afghanistan showed that low HL was significantly higher among older women [28]. This discrepancy may be attributed to the historical context and the drastic changes that have recently taken place in Afghan women’s access to education. While girls were officially banned from schools in the 1990s, school attendance has increased considerably since 2001 [29].

The current study also showed that limited HL was more common among less educated women. People with lower educational attainment have difficulty understanding and using health information and instructions, which contributes to their low HL. Similarly, studies in Iran [24, 30, 31], Japan [32], and the UK [14] confirm this relationship. However, the fact of a high level of education does not always imply a high level of HL. HL involves steps apart from reading and writing, such as understanding complex information, being able to use technology, seeking information, and interpreting acquired knowledge. Other studies revealed that the HL level of Turkish pregnant women is not associated with their educational level [23, 33].

The current research revealed a significant association between women HL level and husband educational level where the risk of limited HL was higher with lower husband education. This relationship may lie in spouses’ role towards each other and educational effects that they achieve from one another. The high level of husband’s literacy increases mother’s awareness and trains her during the married life. Similar results were reported in Iran where the level of women HL got higher along with higher husband education [31, 34].

This study shows that limited HL was more common among housewives. Similar results were reported in Turkey and Afghanistan [23, 28]. In general, employment status logically implies superior socioeconomic level, accompanied by higher HL. On the other hand, a study in Iran found no significant differences between housewives and working women in relation to their HL level, which may be due to social differences between societies [34].

This study indicated a significant relationship between family income and women’s HL level, such that limited HL was more common among women who reported insufficient family income. This relationship can be explained by the limited accessibility of education and treatment services to those with a low salary and poor economic and social conditions. This finding is supported by research in Japan and Iran [32, 34], as well as by work in China [35].

This research found a higher proportion of limited HL among women who do not frequently use the Internet as a source for health information. That is, HL involves steps apart from reading and writing, and using the Internet helps people develop HL. This finding is in harmony with the report of Astantekin et al. that pregnant women’s HL score was lower among those who did not use the Internet for health-related research [33]. Similarly, another study in the UK reported that individuals with no access to the Internet were more likely to have low HL than those who had access [14].

This study found that limited HL is more common among women who either married or had their first child at young age. In fact, women with lower levels of education and lower incomes tend to marry and become pregnant at younger ages, neglecting the consequences of such practices. These results are in harmony with the findings of Guler et al. in Turkey [23].

This study identified a significant relationship between the HL level of pregnant women and pregnancy planning. It was found that limited HL was more common among women with unplanned pregnancies. Women with low HL tend to be unaware of the importance of contraceptive methods, resulting in higher rates of unplanned pregnancy. Similarly, it was reported in Afghanistan that most women with inadequate HL do not know how to prevent unwanted pregnancy [28]. However, the study conducted by Safaie et al. in Iran found no association between pregnancy planning and HL level of women [34]. This divergence can be explained by the high contraceptive prevalence rate (CPR) among Iranian married women (81%) and low unmet need for family planning (5%), which is far different from the figures for Egypt (61% for CPR and 12% for unmet need) [36].

The current study showed that limited HL was more prevalent among women with no history of abortion. It is expected that the experience of abortion may lead women to become more interested in gaining information about abortion and healthy pregnancy, as well as improving their HL. However, this finding is not consistent with the results of some Iranian studies that reported no significant association between abortion history and HL level; here, different social dimensions may play a role [24, 30].

This study revealed that the majority of participants who reported health problems during previous pregnancies had limited HL level. It is expected as those with low HL often find verbal and written health information difficult to comprehend and have a poorer chance of following health guidelines, therefore becoming be more susceptible to health problems. This result is consistent with what was reported in Nigeria, which found a significant association between maternal HL and healthy pregnancy [37]. Furthermore, another study in Iran indicated that low HL was related to low glycemic control in pregnant women [24].

Logistic regression analysis in this research showed that family income (unsatisfactory), educational attainment (lower than university education), and pregnancy planning (unplanned) were significant predictors of limited HL. These results are logical and could be explained by the fact that women with lower levels of education usually find verbal and written health information difficult to comprehend. In addition, they lack skill in making appropriate decisions regarding health issues, and they must deal with this challenge throughout their lives. Usually, women with low educational level have low access to adequate working opportunities, so they either become housewives or work in occupations with low salaries. In both conditions, women suffer from unsatisfactory incomes and lack sufficient financial means to have access to appropriate health services and may seek health advice from untrusted sources, which plays a vital role in shaping their HL. Moreover, women with a low level of HL tend to have limited knowledge of and access to appropriate contraception, as well as having a higher frequency of unplanned pregnancies.

Predictors for problematic HL were studied by Pirdehghan et al. in Iran, and it was found that academic education was a protective factor, while being a housewife was a risk factor. However, this study found that age was not a significant predictor for HL [24]. Another study, conducted in Thailand, reported family income and social support among the significant predictors for women’s HL, while age and educational level were not significant predictors [38].

### Study limitations

This study identified the level of HL and its associated demographic and reproductive factors in pregnant women attending governmental health facilities, so its results cannot be generalized to the all pregnant women. Further studies are recommended to evaluate HL in women attending nongovernmental hospitals and clinics. Another limitation was that, due to the cross-sectional method applied, the research outcomes can only be described as associations, and causation could not be proven.

## Conclusion

This study provided evidence of limited HL and its associated characteristics in pregnant women. Approximately, three-quarters of participating pregnant women were found to have limited HL. The problem was more serious in less educated women and those with insufficient income. In addition, women with unplanned pregnancies were more likely to have limited HL. This deficit must be addressed by health planners and policymakers who are responsible for promoting health and reducing health disparities in the community. Comprehensive efforts are needed to enhance maternal HL, especially in low-income and lower-educated communities. Some practical interventions can help to improve HL, such as clear communication with mothers, application of visual teaching aids, use of understandable and illustrated media, and creation of an enabling environment for questioning and decision-making.

## Data Availability

The datasets used and/or analyzed during this study are available from the corresponding author on reasonable request.

## Abbreviations

HL: Health literacy
ANC: Antenatal care
PHC: Primary health care
MUH: Mansoura University Hospital
HLS-EU-Q16: European Health Literacy Survey Questionnaire-short version
CPR: Contraceptive prevalence rate
